# FFA-ROS-P53-mediated mitochondrial apoptosis contributes to reduction of osteoblastogenesis and bone mass in type 2 diabetes mellitus

**DOI:** 10.1038/srep12724

**Published:** 2015-07-31

**Authors:** Jun Li, Wang He, Bo Liao, Jingyue Yang

**Affiliations:** 1 Department of Orthopaedic Surgery, Tangdu Hospital, Fourth Military Medical University, Xi’an 710038, China; 2 Department of Endocrinology, Xi’an NO.1 Hospital, Xi’an 710002, China; 3 Department of Orthopaedic Surgery, Tangdu Hospital, Fourth Military Medical University, Xi’an 710038, China; 4 Jingyue Yang, Department of Oncology, Xijing Hospital, Fourth Military Medical University, Xi’an 710032, China

## Abstract

This study evaluated the association between free fatty acid (FFA), ROS generation, mitochondrial dysfunction and bone mineral density (BMD) in type 2 diabetic patients and investigated the molecular mechanism. db/db and high fat (HF)-fed mice were treated by Etomoxir, an inhibitor of CPT1, MitoQ, and PFT-α, an inhibitor of P53. Bone metabolic factors were assessed and BMSCs were isolated and induced to osteogenic differentiation. FFA, lipid peroxidation and mtDNA copy number were correlated with BMD in T2DM patients. Etomoxir, MitoQ and PFT-α significantly inhibited the decrease of BMD and bone breaking strength in db/db and HF-fed mice and suppressed the reduction of BMSCs-differentiated osteoblasts. Etomoxir and MitoQ, but not PFT-α, inhibited the increase of mitochondrial ROS generation in db/db and HF-fed mice and osteoblasts. In addition, Etomoxir, MitoQ and PFT-α significantly inhibited mitochondrial dysfunction in osteoblasts. Moreover, mitochondrial apoptosis was activated in osteoblasts derived from db/db and HF-fed mice, which was inhibited by Etomoxir, MitoQ and PFT-α. Furthermore, mitochondrial accumulation of P53 recruited Bax and initiated molecular events of apoptotic events. These results demonstrated that fatty acid oxidation resulted in ROS generation, activating P53/Bax-mediated mitochondrial apoptosis, leading to reduction of osteogenic differentiation and bone loss in T2DM.

Type 2 diabetes mellitus (T2DM) is dramatically increasing in the whole world, resulting in the increase of patients who suffer from various diabetic complications^1^. Diabetic complications can severely lower the quality of life in those patients and rise global medical costs. Diabetes may result in skeletal complication, also called diabetic bone disease, which is characterized by decreased linear bone growth in adolescents, increased risk of osteopenia, osteoporosis and fracture, and impaired potential of bone regeneration^2^. Both type 1 and type 2 diabetes are associated with metabolic abnormalities of bone and bone loss^3^,^4^. Osteoporosis is the most common diabetes-associated metabolic abnormality of bone that is characterized by bone loss, reduction of bone mineral density (BMD) and progressive deterioration of bone microstructure, increased bone fragility and risk of fracture^5^.

Dyslipidemia is one of the hallmarks of T2DM, which contributes to various diabetic complications^6^. Lipid profile was found to be strictly related to bone mass in both men and women^7^. Fat mass is negatively correlated with bone mass when the mechanical loading effect of body weight is statistically removed^8^. Obesity and ectopic accumulation of fat in bone marrow result in decrease of osteoblastogenesis^9^. Moreover, age-related fat accumulation in bone marrow and decrease of osteoblast differentiation *in vivo* are related with increased levels of free fatty acid (FFA) oxidation (FAO)^10^.

It is believed that oxidative stress contributes to the pathogenesis and development of diabetes^11^. Moreover, oxidative stress is recognized as a crucial initiating factor for impaired osteoblastic bone formation in osteoporosis^12^. Using a co-culture system *in vitro*, Dong *et al.* identified that FFA released by the adipocytes inhibited osteoblasts proliferation and function and induced osteoblasts apoptosis through generation of reactive oxygen species (ROS)^13^. However, the *in vivo* correlation between dyslipidemia, ROS generation and bone mass in T2DM is still unknown. The mechanism of FFA-mediated inhibition of osteoblasts function is far from completely understood.

The present study was designed to (1) investigate correlation between FFA, ROS generation and bone mass in T2DM patients; (2) elucidate the signaling pathway responsible for reduction of bone mass *in vivo* under T2DM conditions. We identified that circulating levels of FFA, lipid peroxidation and mtDNA copy number were correlated with BMD in T2DM patients. We suggested that in db/db and high fat (HF) diet-fed mice, fatty acid oxidation resulted in ROS generation, activating P53/Bax-mediated mitochondrial dysfunction and apoptosis, leading to the reduction of osteogenic differentiation and bone loss.

## Results

### Association between FFA, FBG, oxidative stress, mtDNA copy number and BMD in T2DM patients

Forty-six patients of T2DM were included in the study. To exclude the possible interference of medication, the patients were newly diagnosed. To distinguish with the decrease of BMD in postmenopausal women, only male T2DM patients were included. Mean age was 50.6 ± 12.5 years. Mean body mass index (BMI) was 24.8 ± 2.8 kg/m^2^. Mean fasting blood glucose (FBG) was 10.5 ± 2.2 mmol/L. Mean free fatty acid (FFA) was 0.69 ± 0.14 mmol/L (Table 1).

**Figure 1:**
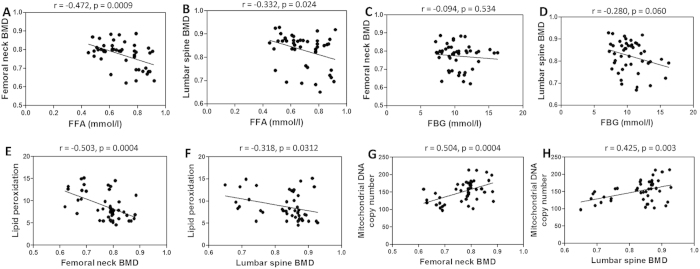
Correlation between FFA, FBG, lipid peroxidation, mtDNA copy number and BMD in T2DM patients. (**A**) Correlation between femoral neck BMD and FFA. (**B**) Correlation between lumbar spine BMD and FFA. (**C**) Correlation between femoral neck BMD and FBG. (**D**) Correlation between lumbar spine BMD and FBG. (**E**) Correlation between lipid peroxidation and femoral neck BMD. (**F**) Correlation between lipid peroxidation and lumbar spine BMD. (**G**) Correlation between mtDNA copy number and femoral neck BMD. (**H**) Correlation between mtDNA copy number and lumbar spine BMD.

In the study, we evaluated the relationship between circulating levels of FFA, FBG, oxidative stress, mtDNA copy number and BMD in T2DM patients. As shown in Fig. 1A,B, both femoral neck and lumbar spine BMD were negatively correlated with FFA level (r = −0.472, p = 0.0009; r = −0.332, p = 0.024), respectively. In constrast, both femoral neck and lumbar spine BMD were not significantly correlated with glucose level (r = −0.094, p = 0.534; r = −0.280, p = 0.060) (Fig. 1C,D). We proposed that FFA, but not glucose, contributed to bone loss in T2DM patients. To assess the level of oxidative stress, lipid peroxidation in peripheral blood mononuclear cells (PBMCs) from T2DM patients were analyzed. Lipid peroxidation was negatively correlated with femoral neck BMD (r = −0.503, p = 0.0004) and lumbar spine BMD (r = −0.318, p = 0.0312) (Fig. 1E,F). It was well-known that mitochondrial dysfunction was usually associated with the generation of ROS in mitochondria, leading to oxidative stress. In our study, we also measured mtDNA copy number in PBMCs and investigated the relationship between mtDNA copy number and BMD in T2DM patients. As shown in Fig. 1G,H, mtDNA copy number was positively correlated with femoral neck BMD (r = 0.504, p = 0.0004) and lumbar spine BMD (r = 0.425, p = 0.003).

Table 2 showed the multivariate linear regression analyses involving all of the above parameters, with BMD as a dependent variable. Femoral neck and lumbar spine BMD were inversely associated with FFA, lipid peroxidation and BMI, and positively associated with mtDNA. FBG and age had no significant effect on BMD. The multivariate linear regression analysis showed an independent and inverse association of FFA, lipid peroxidation and BMI, and positive association of mtDNA with BMD in type 2 diabetic patients.

### Alterations of serum biochemical factors, BMD and bone breaking force in mice and osteogenic differentiation in isolated BMSCs

The above results of clinical studies showed that elevated FFA levels, oxidative stress, and mitochondrial dysfunction were closely related with reduction of BMD in T2DM. In addition, our data and literature indicated that activation of p53 was involved in the decrease of osteoblasts under certain conditions^14^. In the present study, most of the experiments were conducted in db/db obese and diabetic mice. In addition, to support the investigation of effect of lipid metabolic disorder on bone metabolism, C57BL/6 mice were given high fat (HF) diet to induce lipid accumulation. db/db and HF diet-fed mice were treated with Etomoxir, an inhibitor of carnitine palmi-toyltransferase 1 (CPT1) which was a rate-limiting enzyme for the conversion of long chain acyl-CoA to long chain acylcarnitine, mitoquinone (MitoQ), a mitochondria-targeted antioxidant, and PFT-α, a P53 inhibitor.

After the treatment, serum FBG, and lipid profiles were determined in db/db mice. As shown in Table 3, MitoQ significantly reduced the level of FBG in db/db mice. Etomoxir and PFT-α had no remarkable effect on FBG in db/db mice. MitoQ markedly inhibited the changes of lipid profiles, including the increase of FFA, TG, and LDL, and the decrease of HDL. The profiles of lipids in db/db mice were not altered by Etomoxir and PFT-α. The results demonstrated that disorder of lipid metabolism occurred in db/db diabetic mice and MitoQ, but not Etomoxir and PFT-α, could improve lipid metabolism.

**Figure 2:**
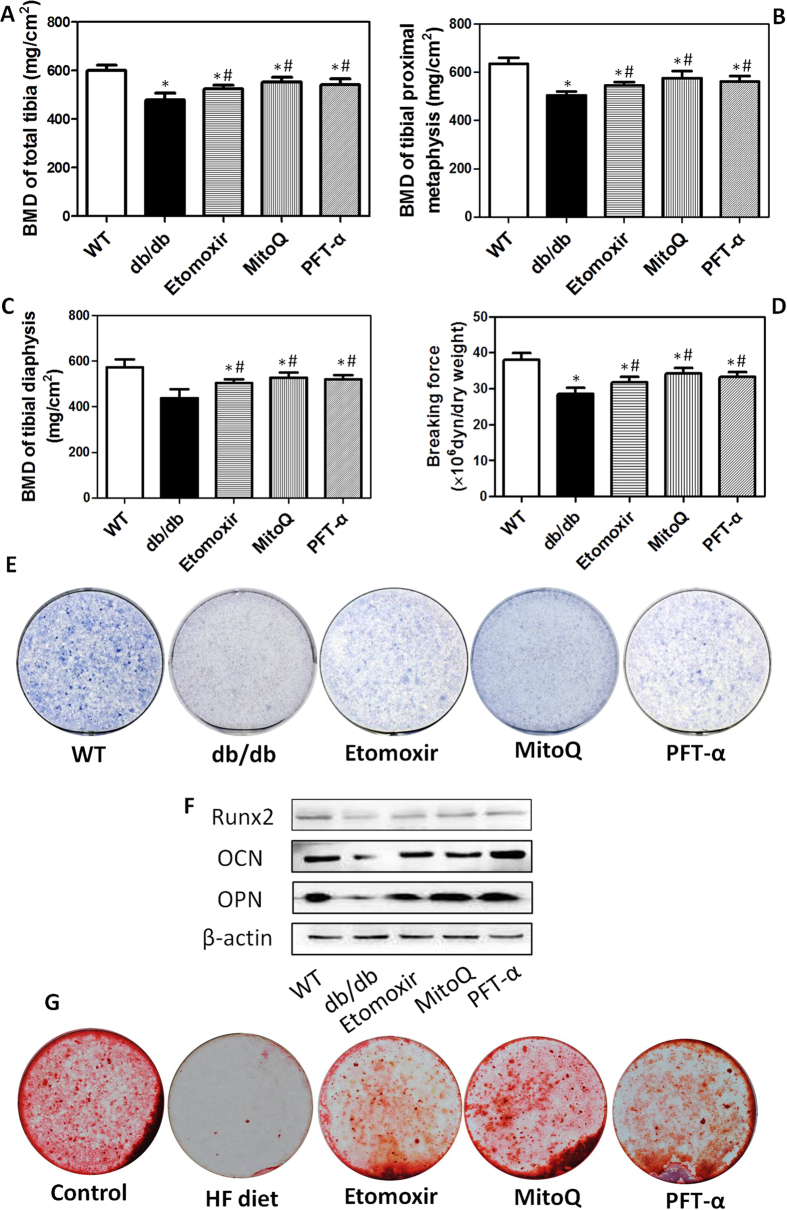
Alterations of bone mass in mice and osteogenic differentiation of BMSCs *in vitro.* db/db mice were administered with Etomoxir, an inhibitor of CPT1, MitoQ, a mitochondrial specific antioxidant, and PFT-α, an inhibitor of P53, for 8 weeks. After that, bone mass density of total tibia (**A**), tibial proximal metaphysic (**B**) and tibial diaphysis (**C**), and breaking strength (**D**) were measured. BMSCs were isolated and induced to differentiate into osteoblast. Then, osteogenic differentiation was evaluated by alkaline phosphatase staining (**E**). Protein expression of Runx2, OCN and OPN in differentiated osteoblasts was detected by western blot (**F**). BMSCs isolated from C57BL/6 mice were induced to differentiate into osteoblast in the presence or absence of 250 μM palmitate with or without 10 μM Etomoxir, 100 nM MitoQ, 10 μM PFT-α. Then, osteogenic differentiation was evaluated by alizarin red staining (**G**). *p < 0.05, compared with WT. ^#^p < 0.05, compared with db/db mice.

In the next step, serum bone metabolic factors, BMD and bone breaking force were evaluated. Serum alkaline phosphatase (ALP), a byproduct of osteoblast activity, was increased in db/db mice, which event was significantly suppressed by Etomoxir, MitoQ and PFT-α (Table 3). In addition, serum level of osteocalcin (OCN), a marker of bone formation, was reduced in db/db mice and Etomoxir, MitoQ and PFT-α markedly inhibited the reduction of OCN (Table 3). Moreover, serum tartrate-resistant acid phosphatase (TRAP), a marker of bone resorption, was elevated in db/db mice which phenomenon was significantly suppressed by Etomoxir, MitoQ and PFT-α (Table 3). Tibial BMD was assessed by a DXA for small animals. As shown in Fig. 2A–C, BMD of total tibia, tibial proximal metaphoysis and tibial diaphysis were decreased in db/db mice. In supplemental Fig. 1A, we showed that BMD of total tibia in mice fed HF diet were decreased significantly. As respected, Etomoxir, MitoQ and PFT-α could significantly inhibit the decrease of tibial BMD (Fig. 2A–C and supplemental Fig. 1A). Concomitantly, bone breaking strength was down-regulated in db/db mice, which could be evidently suppressed by Etomoxir, MitoQ and PFT-α (Fig. 2D). These results demonstrated that FFA metabolism in mitochondria, mitochondrial ROS generation and P53 activation were involved in the reduction of BMD in db/db mice.

To evaluate the effect of Etomoxir, MitoQ and PFT-α on osteogenic differentiation, BMSCs were isolated and induced to differentiation *in vitro.* In the study, we used the nomination “osteogenic differentiation_(db)_” for the osteoblasts differentiated from BMSCs of db/db mice and used the nomination “osteogenic differentiation_(HF)_” for the osteoblasts differentiated from BMSCs of HF diet-fed mice. As shown in Fig. 2E, osteogenic differentiation of BMSCs was determined by ALP staining. Osteogenic differentiation_(db)_ was markedly decreased, as reflected by reduction of ALP staining (Fig. 2E). Osteogenic differentiation_(HF)_ was also significantly decreased, as reflected by reduction of alizarin red staining (supplemental Fig. 1B). In db/db mice and HF diet-fed mice treated by Etomoxir, MitoQ and PFT-α, osteogenic differentiation of BMSCs was markedly increased (Fig. 2E and supplemental Fig. 1B). Moreover, in differentiated osteoblasts, protein expression of Runt-related transcription factor (Runx2), a key transcription factor associated with osteoblast differentiation, OCN, and osteopontin (OPN) which played a role in osteoclast adhesion to bone, were down-regulated (Fig. 2F). In db/db mice, Etomoxir, MitoQ and PFT-α treatment could significantly increase Runx2, OCN and OPN protein expression (Fig. 2F). To evaluate the direct effect of FFA on osteoblast differentiation, BMSCs were isolated from C57BL/6 mice and induced to differentiation in the presence or absence of palmitate, the most abundant form of FFA in circulation, with or without Etomoxir, MitoQ and PFT-α. As shown in Fig. 2G, palmitate significantly inhibited alizarin red staining which was notably blocked by Etomoxir, MitoQ and PFT-α, indicating that FFA could inhibit osteogenic differentiation which involved FAO, ROS generation and P53 activation. The data indicated that decreased osteogenic differentiation was involved in the reduction of BMD in mice and FFA metabolism in mitochondria, mitochondrial ROS generation and P53 activation were responsible for the inhibition of osteoblast differentiation.

### Alterations of oxidative stress-related markers in mice and differentiated osteoblasts *in vitro*

**Figure 3:**
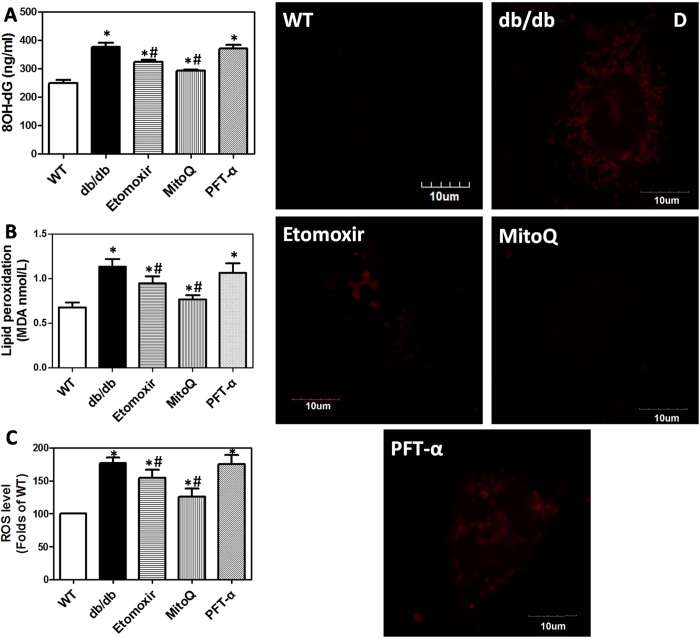
Alterations of oxidative stress-related markers in urine and PBMCs in mice and in differentiated osteoblast *in vitro.* db/db mice were administered with Etomoxir, an inhibitor of CPT1, MitoQ, a mitochondrial specific antioxidant, and PFT-α, an inhibitor of P53, for 8 weeks. After that, 8OH-dG (**A**) in urine and lipid peroxidation (**B**) in PBMCs were determined by commercial kits. Then, osteogenic differentiation of BMSCs was induced and intracellular ROS level was examined by DCFH-DA (**C**). Mitochondrial superoxide anion in differentiated osteoblasts was determined by staining of MitoSOX (**D**), a mitochondrial superoxide anion specific probe. *p < 0.05, compared with WT. ^#^p < 0.05, compared with db/db mice.

To confirm the role of ROS in T2DM-associated reduction of BMD and whether ROS generation was upstream or downstream of FFA metabolism in mitochondria and P53 activation, we evaluated oxidative stress-related markers. As shown in Fig. 3A and supplemental Fig. 2, urine 8-hydroxy-2′- deoxyguanosine (8OH-dG), a marker of DNA oxidative damage, was increased in db/db mice and HF diet-fed mice. The administration of Etomoxir and MitoQ, but not PFT-α, significantly decreased urine 8OH-dG level in db/db mice and HF diet-fed mice. Consistently, lipid peroxidation in PBMCs was elevated in db/db mice, which event was significantly inhibited by the treatment of Etomoxir and MitoQ, but not PFT-α (Fig. 3B). DCFH-DA, an oxidation-sensitive probe was used to detect intracellular ROS level in differentiated osteoblasts. As shown in Fig. 3C, intracellular ROS level in osteoblasts_(db)_ was enhanced. Etomoxir and MitoQ significantly inhibited the increase of ROS generation in osteoblasts_(db)_, as evidenced by reduction of DCF fluorescence (Fig. 3C). Moreover, MitoSOX was used to detect mitochondria-specific superoxide anion generation. In Fig. 3D, we showed that in osteoblasts_(db)_, MitoSOX fluorescence was strongly increased. Etomoxir and MitoQ could remarkably inhibit the increase of MitoSOX staining (Fig. 3D). PFT-α had no significant effect on both intracellular and mitochondrial ROS generation (Fig. 3C and D). These results demonstrated that FFA metabolism in mitochondria was involved in mitochondrial ROS generation which contributed to the reduction of BMD and osteogenic differentiation under T2DM conditions. These results also implicated that P53 activation was not involved in mitochondrial ROS generation in T2DM.

### Alterations of mitochondrial function in differentiated osteoblasts derived from BMSCs in mice

**Figure 4:**
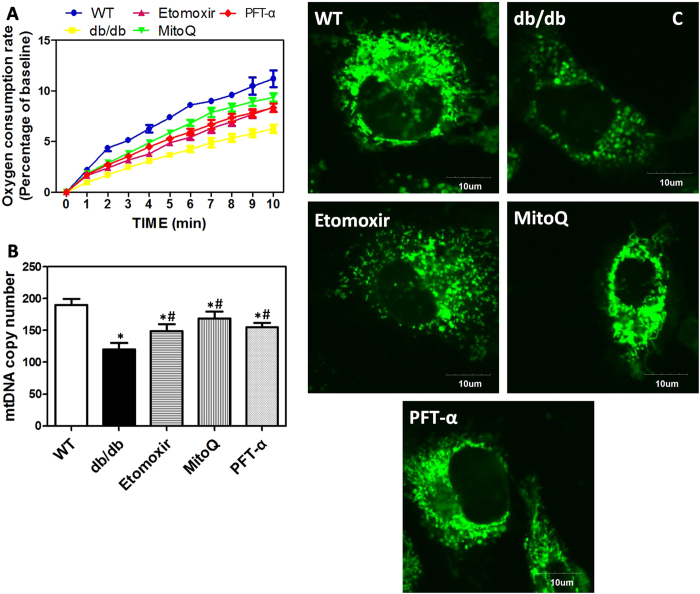
Alterations of mitochondrial function in differentiated osteoblasts *in vitro.* db/db mice were administered with Etomoxir, an inhibitor of CPT1, MitoQ, a mitochondrial specific antioxidant, and PFT-α, an inhibitor of P53, for 8 weeks. After that, osteogenic differentiation of BMSCs was induced *in vitro.* Oxygen consumption rate was evaluated by a Clark Clark Oxygen Electrode (**A**). mtDNA copy number was detected by Real-time PCR (**B**). Mitochondrial membrane potential was measured by Rho123 (**C**). *p < 0.05, compared with WT. ^#^p < 0.05, compared with db/db mice.

Next, we evaluated mitochondrial function through determining oxygen consumption rate, mtDNA copy number, and mitochondrial membrane potential (MMP) in differentiated osteoblasts derived from BMSCs in db/db mice and HF diet-fed mice. In Fig. 4A, we showed that oxygen consumption rate in osteoblasts_(db)_ declined significantly. Etomoxir, MitoQ and PFT-α treatment notably blocked the decrease of oxygen consumption rate (Fig. 4A). In addition, in osteoblasts_(db)_ and osteoblasts_(HF)_, mtDNA copy number was decreased to nearly half of that in wild type (WT) and Control mice (Fig. 4B and supplemental Fig. 3). In osteoblasts_(db)_ and osteoblasts_(HF)_ treated by Etomoxir, MitoQ and PFT-α, mtDNA copy number was significantly increased, compared with that of db/db mice and HF diet-fed mice (Fig. 4B and supplemental Fig. 3). Moreover, Rho123 was used to assess the changes of MMP in the study. As illustrated in Fig. 4C, the fluorescence intensity in osteoblasts_(db)_ was notably lower than that of WT mice. Etomoxir, MitoQ and PFT-α treatment notably blocked the decrease of MMP in differentiated osteoblasts. The results demonstrated that during differentiation of osteoblasts from BMSCs, FFA metabolism in mitochondria, mitochondrial ROS generation and P53 activation resulted in the reduction of mitochondrial dysfunction.

### Alterations of apoptosis and its related factors in differentiated osteoblasts derived from BMSCs in db/db mice

**Figure 5:**
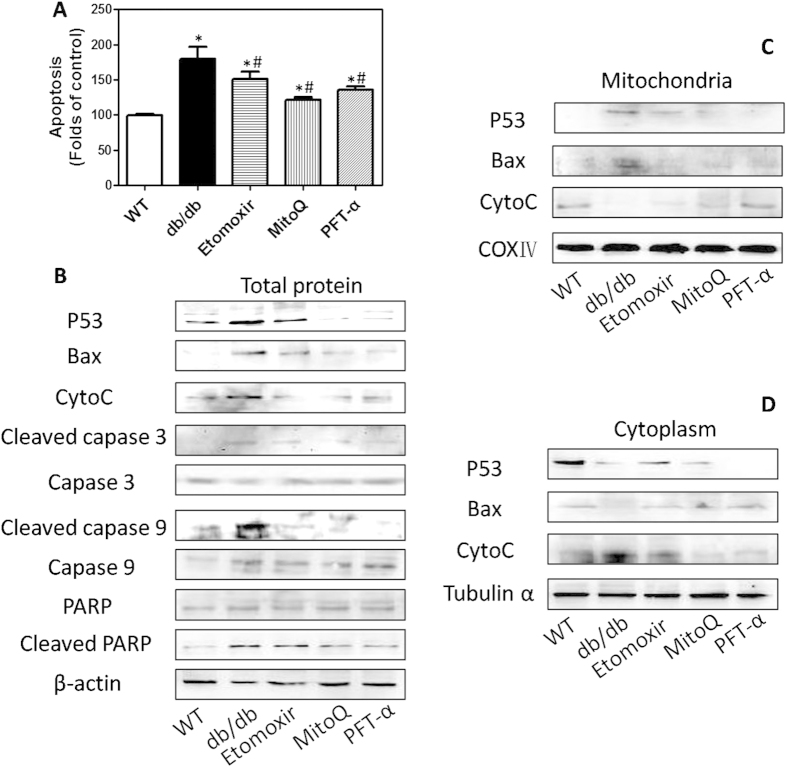
Alterations of apoptosis-related factors in differentiated osteoblasts *in vitro.* db/db mice were administered with Etomoxir, an inhibitor of CPT1, MitoQ, a mitochondrial specific antioxidant, and PFT-α, an inhibitor of P53, for 8 weeks. After that, osteogenic differentiation of BMSCs was induced *in vitro.* TUNEL assay was conducted to evaluate apoptosis (**A**). Total expression (**B**), mitochondrial (**C**) and cytoplasmic expression (**D**) of apoptosis-related markers were determined by western blot.

In the next step, we measured apoptosis and related key factors in osteoblasts_(db)_ and osteoblasts_(HF)_. As illustrated in Fig. 5A and supplemental Fig. 4, percentage of TUNEL-positive cells were calculated and results were expressed as percentage of apoptotic cells in WT or Control mice. Osteoblasts_(db)_ and osteoblasts_(HF)_ exhibited high level of apoptosis. In mice treated by Etomoxir, MitoQ and PFT-α, apoptosis in BMSCs-differentiated osteoblasts was significantly suppressed, as reflected by decrease of TUNEL-positive cells (Fig. 5A and supplemental Fig. 4). In Fig. 5B, we showed that under diabetic conditions, the protein expression of P53, Bax and cytochrome c (CytoC), and the cleavage of caspase 3, caspase 9 and poly-ADP-ribose polymerase (PARP) in osteoblasts_(db)_ were evidently elevated. In Etomoxir, MitoQ and PFT-α-treated mice, these alterations were significantly inhibited (Fig. 5B). To further explore the molecular mechanism, we isolated mitochondrial and cytoplasmic fractions and evaluated the molecular events. As shown in Fig. 5C, in osteoblasts_(db)_, P53 and Bax proteins in mitochondria was increased, and CytoC in mitochondria was decreased. In osteoblasts_(db)_ treated by Etomoxir, MitoQ and PFT-α, the accumulation of P53 and Bax and the release of CytoC were significantly inhibited (Fig. 5C). In cytoplasm, P53 and Bax protein expression was decreased and CytoC content was increased, which event was blocked by Etomoxir and MitoQ. In PFT-α-treated diabetic mice, the decrease of Bax and increase of CytoC were also inhibited. The results demonstrated that during differentiation of osteoblasts from BMSCs FFA metabolism in mitochondria, mitochondrial ROS generation and P53 activated mitochondrial apoptotic cascades, resulting in apoptosis. FFA metabolism and mitochondrial ROS generation were involved in the activation and mitochondrial translocation of P53, which was responsible for the activation of mitochondrial apoptotic pathway.

### P53 and Bax interactions in differentiated osteoblasts derived from BMSCs in db/db mice

**Figure 6:**
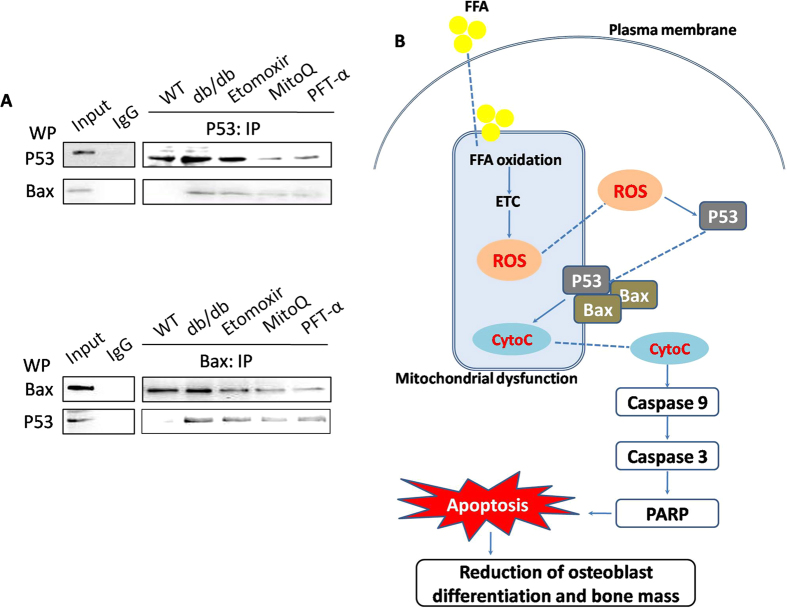
Protein interaction of P53 and Bax in differentiated osteoblasts *in vitro.* db/db mice were administered with Etomoxir, an inhibitor of CPT1, MitoQ, a mitochondrial specific antioxidant, and PFT-α, an inhibitor of P53, for 8 weeks. After that, osteogenic differentiation of BMSCs was induced *in vitro.* Co-IP was conducted to detect the protein:protein interaction between P53 and Bax (**A**). (**B**) Schematic figure of the potential molecular mechanism of FFA-mediated reduction of osteogenic differentiation and bone mass under type 2 diabetic conditions.

Since both P53 and Bax accumulated in mitochondria in osteoblasts_(db)_, we evaluated possible interactions between these two key proteins in the next step. As shown in Fig. 6A, in osteoblasts_(db)_, Bax antibody recognized a discrete band in P53-immunoprecipitated material and P53 antibody recognized a discrete band in Bax-immunoprecipitated material, indicating the interaction between P53 and Bax under diabetic conditions. In osteoblasts_(db)_ treated by Etomoxir, MitoQ and PFT-α, P53 and Bax interaction was inhibited, indicating that FFA metabolism and mitochondrial ROS generation were involved in the activation of P53 and Bax interaction.

## Discussion

T2DM is closely associated with the occurrence of bone loss and various bone metabolic disorders. However, the mechanism of diabetes-related reduction of bone mass is not clear and clinical treatment for diabetic bone disease is limited. In the present study, we investigated the relationship between circulating FFA, glucose, lipid peroxidation, mtDNA copy number and BMD in T2DM patients and elucidated the molecular mechanism underlying T2DM-associated reduction of bone mass. Our results showed that circulating levels of FFA, lipid peroxidation and mtDNA copy number, but not glucose, were correlated with BMD in T2DM patients, indicating that elevated FFA, oxidative stress and mitochondrial dysfunction may be involved in T2DM-associated bone loss.

FFA undertakes the process of FAO in mitochondria in which FFA could be broken down to produce energy. CPT1 is a rate-limiting enzyme for the conversion of long chain acyl-CoA to long chain acylcarnitine, which is important for the transportation of FFA across the inner mitochondrial membrane to be oxidized. The metabolism of FFA may generate acetyl-CoA, a substrate for trichloroacetic acid (TCA) cycle. The reducing equivalent NADH and FADH2 generated in FAO and TCA cycle supply excessive electrons for mitochondrial oxidative phosphorylation (OXPHOS)^15^, resulting in increased production of ROS as by-products^16^. To elucidate the sequence of FFA elevation, oxidative stress and mitochondrial dysfunction and study the molecular mechanism of these events, db/db obese and diabetic mice and HF diet-fed mice were introduced and treated by Etomoxir, an inhibitor of FFA oxidation-related key enzyme CPT1, and MitoQ, a mitochondria-targeted antioxidant, respectively. In addition, our data and literature have demonstrated that P53 may play a role in bone loss under pathological conditions^14^. In the current study, we also administrated mice with PFT-α, an inhibitor of P53.

The results showed that Etomoxir, MitoQ and PFT-α could notably inhibit bone loss in db/db and HF diet-fed mice. Moreover, BMSCs in db/db were isolated and induced to osteogenic differentiation. In groups treated by Etomoxir, MitoQ and PFT-α, osteogenic differentiation was significantly improved, indicating that FAO, mitochodnrial ROS generation and P53 activation were involved in the reduction of osteogenic differentiation, which played a role, at least partly, in T2DM-associated bone loss. Etomoxir and MitoQ, but PFT-α, could decrease oxidative stress-related markers in db/db mice and ROS level in BMSCs-differentiated osteoblasts, indicating that FAO was responsible for mitochondrial ROS generation under the present experimental conditions.

In a cell, the release of ROS from mitochondria is obligatory and varies with the redox state of the electron carriers according to the mitochondrial oxidative metabolism^17^,^18^. In turn, mitochondria are the main target of ROS insult. Mitochondrial DNA (mtDNA) deletion was reported to be associated oxidative stress and severe male osteoporosis^19^. Moreover, mtDNA copy number in peripheral blood was associated with femoral neck BMD in postmenopausal women^20^. In our study, we found that mitochondrial function was decreased in BMSCs-differentiated osteoblasts from db/db and HF diet-fed mice, as reflected by decreased oxygen consumption rate, decreased mtDNA copy number and decreased MMP. Etomoxir, MitoQ and PFT-α could notably inhibit mitochondrial dysfunction, implicating that FAO-induced mitochondrial ROS generation participated in the induction of mitochondrial dysfunction and P53 may be a mediator of ROS-exerted effect on mitochondrial function.

ROS is believed to function as critical regulators of apoptosis^21^,^22^. Either exogenously administered or endogenously produced ROS, could induce the opening of the mitochondrial permeability transition pore and causes apoptosis^21^,^23^. Apoptosis is involved in numerous pathophysiological conditions, including the inhibition of osteogenic differentiation and bone loss^24^,^25^. Mitochondria play an important role in apoptosis, through releasing a number of proapoptotic factors to cytosol, including CytoC, aif, smac/diablo, and endoG. These factors in turn activate a variety of enzymatic executors of apoptosis, leading to cleavage of proteins and DNA. It has been shown that in response to apoptotic signals, proapoptotic factors such as Bax translocates to the outer mitochondrial membrane where they form channels and/or regulate the function of preexisting channels, leading to disruption of mitochondrial membrane integrity^26^. In the study, we showed that in BMSCs-differentiated osteoblasts from db/db and and HF diet-fed mice, mitochondrial apoptotic pathway was activated and apoptosis was increased. Moreover, mitochondrial accumulation of P53 of Bax was induced in osteoblasts_(db)_. Inhibition of P53 by PFT-α could suppress Bax translocation to mitochondria, the activation of apoptotic cascades and final apoptosis, indicating that P53 activation recruited Bax to mitochondria and activated downstream apoptotic cascades, leading to apoptosis in BMSCs-differentiated osteoblasts. Furthermore, Etomoxir and MitoQ inhibited mitochondrial expression of P53 and Bax, and the initiation of apoptotic events, indicating that FAO-generated ROS was responsible for P53-regulated apoptosis. To detect the direct interaction between P53 and Bax, Co-IP assay was introduced in the study. We found that in db/db mice, P53 interacted directly with Bax, which was inhibited by Etomoxir, MitoQ and PFT-α, further confirming the direct interaction of P53 and Bax, and the involvement of FAO-mediated mitochondrial ROS generation.

In conclusion, the main and novel finding of this study was that in T2DM, elevated FFA resulted in mitochondrial ROS generation, which activated P53/Bax interaction, leading to mitochondrial dysfunction, apoptosis, and the reduction of osteogenic differentiation and bone loss (Fig. 6B). Despite of a direct relation between FFA/oxidative stress and osteoblast function and survival, other indirect effects may be involved in the reduction of osteogenic differentiation. For example, changes in paracrine or cell communication (between osteoblasts and adipocytes, marrow cells, immune cells and osteoclasts) *in vivo* may also occur under oxidative stress and might contribute to the reversion of the low osteogenic status observed with the treatments. All these assumptions should be examined in future study. Overall, our findings suggest that redox state is representative of metabolic status in patients with T2DM. The implication has appointed a new path toward the understanding of pathogenesis and therapy of osteoporosis in T2DM patients.

## Materials and Methods

### Chemicals and reagents

P53, Caspase 3, Caspase 9, PARP and COXIV antibodies were purchased from Cell Signaling. β-actin, Bax, cytochrome c, Runx2, osteocalcin and osteopontin antibodies were purchased from Santa Cruz Biotechnology. Tubulin antibody was purchased from Bioworld Technology. DCFH-DA was purchased from Sigma. Most of the chemicals and reagents used in this study were procured from Sigma.

### Patients study

This study of patients was a cross-sectional investigation. Data was collected from September 2013 to December 2013. Forty-six male participants during this study period were included in the study. The subjects included were newly diagnosed with T2DM based on fasting blood glucose and glycated haemoglobin levels who attended Department of Endocrinology, Xi’an NO.1 Hospital, and consented to participate in this study. T2DM diagnosis was established by an experienced internist physician according to case definition criteria. Patients’ ages were obtained during face-to-face interviews. Body weight was measured by a digital wheelchair scale, body height was obtained through measuring the supine length, and body mass index (BMI) was calculated as body weight (kilograms) divided by height (meters) squared. Dual-energy Xray absorptiometry (DXA) with a lunar DPX GE medical system was used to assess BMD in femur neck and spinal lumbar vertebras in patients. All scans were performed according to the manufacturer’s guidelines. Peripheral blood samples were taken after 12 h of fasting from all patients for the subsequent detections. The study protocol was approved by the Ethics Committee of Tangdu Hospotal, the Fourth Military Medical University, and the Ethics Committee of Xi’an NO.1 Hospital, according to the principles of the Declaration of Helsinki. All patients signed an informed written consent.

### Animal treatment

All animal experiments were performed according to the procedures approved by Fourth Military Medical University Animal Care and Use Committee and were carried out in accordance with the approved guidelines. 80 male C57BLKS/J lar-Lepr^db/db^ mice and 20 wild type littermates (8 week) were obtained from Model Animal Research Centre, Nanjing University, China. Mice were housed in cages in a limited access room, under temperature (23 ± 2 °C) and humidity (55 ± 5%) condition with a standard light (12 h light/dark) cycle and fed a regular diet. db/db mice were randomly divided into four groups: db/db group, Etomoxir group, MitoQ group, and PFT-α group. In the Etomoxir group, mice were intraperitoneally injected with 1 mg/kg Etomoxir twice every week. In the MitoQ group, 50 μmol/L MitoQ was given to the mice in water. Water bottles, containing either MitoQ, were covered with aluminum foil, and all bottles were refilled every 3 days. In the PFT-α group, mice were intraperitoneally injected with 1 mg/kg PFT-α twice every week. WT mice were administrated with vehicle instead. The experimental period is 8 weeks. At the end, peripheral blood samples and bone marrow cells were harvested for the assays.

100 C57BL/6 mice obtained from Experimental Animal Centre of Fourth Military Medical University. The mice were randomly divided into five groups: Control group, HF diet group, Etomoxir group, MitoQ group, and PFT-α group. Mice in HF diet, Etomoxir, MitoQ, and PFT-α groups were given high fat diet for 20 weeks and mice in Etomoxir, MitoQ, and PFT-α groups were administrated with Etomoxir, MitoQ, and PFT-α in the last 10 weeks. The administration of Etomoxir, MitoQ, and PFT-α were identical to the treatment in db/db mice. Control mice were administrated with vehicle instead.

### Isolation and *in vitro* differentiation of bone marrow stromal cells

Adherent bone marrow stromal cells (BMSCs) were isolated and induced to differentiate into osteoblast as previously reported^27^. In Brief, femurs and tibias from experimental animals were flushed with complete DMEM medium using a 21-gauge needle attached to a 10-mL syringe. Cells were filtered through a 70-micron nylon mesh. Then, cells were incubated in BMSCs growth media at 37 °C with 5% humidified CO_2_. Fresh medium was added every 2 to 3 days to remove nonadherent cells. The BMSCs reaching 80% confluence were defined as BMSCs at passage 0, and were harvested and diluted 1:3 in MSC growth media, plated, and grown to confluence for further expansion. After the third passage, BMSCs could be used for subsequent detection and differentiation-inducing experiments.

To induce osteogenic differentiation, BMSCs were diluted in osteogenic medium (DMEM supplemented with 10% FCS, 0.2 mM dexamethasone, 10 mmol/L β glycerol phosphate, and 50 mg/mL ascorbic acid) and plated in culture dishes. Medium was aspirated and replaced with fresh osteogenic medium every 3 days. After 14 days in culture, cells were washed with PBS, fixed with ethanol, and stained for alkaline phosphatase (ALP) or alizarin red.

To evaluate the direct effect of FFA on osteogenic differentiation of BMSCs, BMSCs were isolated from C57BL/6 mice and induced to osteogenic differentiation in the presence or absence of palmitate with or without indicated inhibitors. After the culture, cells were washed with PBS, fixed with ethanol, and stained for ALP or alizarin red.

### Measurement of BMD

BMD of the whole tibiae were measured by a DXA. Analysis of tibial BMD was conducted as previously described^28^. In brief, the proximal first quintile of the tibia, representing the trabecular sites, and the middle (second and third quintiles) of the tibia, representing the cortical diaphyseal region, were subjected to BMD measurement.

### Femoral mechanical strength

The femoral bone strength at the middle diaphysis was examined by measuring the mechanical strength as previously reported^29^. Briefly, the force necessary to produce a break at the center of the femur was measured according to the following conditions: the sample space was 1.0 cm, the plunger speed was 100.0 mm/min, the load range was 50.0 kg, and the chart speed was 120.0 cm/min. After the test, the femurs were dried at 95 °C for 24 h to measure their dry weight. The results were expressed as breaking force per dry weight of femurs.

### Biochemical measurement

Blood samples were centrifuged at 3000 rpm for 10 minutes at 4 °C. Serum FFA was measured using an ELISA kit (CUSABIO BIOTECH) according to the manufacturer’s instructions. Serum triglyceride (TG), HDL and LDL were detected by commercial assay kits (Nanjing Jiancheng Company, China). Serum alkaline phosphatase (ALP), osteocalcin (OCN) and tartrate-resistant acid phosphatase (TRAP) were measured to assess osteogenic and osteoclastic activity using the Elisa assay kits.

### Measurement of lipid peroxidation

PBMCs were isolated and purified by isopycnic centrifugation using Histopaque-1119 and Histopaque-1077 (Sigma). Lipid peroxidation in PBMCs and differentiated osteoblasts were evaluated by the measurement of thiobarbituric acid-reactive substances (TBARS) levels using a commercial kit according to the manufacturer’s instructions (Cell Biolabs).

### Measurement of 8-hydroxy-2′-deoxyguanosine in urine

After the experiment, urine samples were collected directly from the urinary bladder with a 10 ml syringe after fasting the mice overnight. Urine 8-hydroxy-2′-deoxyguanosine (8OH-dG) was measured in using a commercial kit (Genox) according to the manufacturer’s instructions.

### Measurement of ROS level

ROS level in differentiated osteoblasts was measured by the oxidation-sensitive fluorescent probe DCFH-DA as previously described^30^. As a nonpolar compound, DCFH-DA could be readily diffused into cells, where it is cleaved by intracellular esterases to form DCFH and thus is trapped inside the cells. DCFH could be oxidized by ROS to generate highly fluorescent 2,7-dichlorofluorescein (DCF). In brief, 2 × 10^6^ cultured BMSCs were incubated with 10 μM DCFH-DA at 37 °C for 30 min, and then washed twice with PBS. The intensity of dichlorofluorescein (DCF) fluorescence was measured with a flow cytometry. ROS level was expressed as folds of WT.

### Measurement of mitochondrial superoxide anion

Mitochondrial superoxide anion in differentiated osteoblasts was examined by a mitochondrial-targeted probe, MitoSOX. Briefly, cultured BMSCs were incubated with MitoSOX(500 nM) at 37 °C for 30 min, and then observed under a confocal microscopy (Olympus).

### mtDNA measurements

mtDNA was extracted from whole blood in patients and from differentiated osteoblasts in mice using a commercial kit (Qiagen). Relative mtDNA copy number was measured using real-time polymerase chain reaction with a SYBR Green kit (Pierce). The number of PCR cycles required for 20 ng DNA was defined as the threshold cycle number (Ct), and mtDNA copy number was calculated as follows: relative copy number = 2ΔCt (ΔCt = Ct β-globin – Ct ND1).

### Oxygen consumption analysis

Oxygen consumption in differentiated osteoblasts was analyzed as previously described^31^. Briefly, cultured BMSCs cells were trypsinized, washed in PBS and then resuspended in Dulbecco’s phosphate-buffered saline (dPBS). The rate of oxygen consumption was measured with a Clark Oxygen Electrode. An equal number of cells under each condition were collected to detect the oxygen consumption rate.

### Measurement of mitochondrial membrane potential

Mitochondrial membrane potential (MMP) in differentiated osteoblasts was measured by the retention of rhodamine (Rho) 123, a specific fluorescent cationic dye that is readily sequestered by active mitochondria, depending on their transmembrane potential. Briefly, cultured BMSCs were incubated with Rho123 (10 μM) at 37 °C for 30 min, and then observed under a confocal microscopy (Olympus).

### Western blot

Briefly, cell lysates of differentiated osteoblasts were prepared by incubation on ice with lysis buffer (50 mM Tris-Cl (pH 7.5), 20 mM NaCl, 5 mM EDTA, 1% TX-100, 0.1% SDS, 5% glycerol + protease inhibitors), and centrifuged at 20,000 × g. For the detection of apoptosis-related markers, Mitochondria Isolation Kit (Thermo) was used to isolated mitochondrial protein. Protein concentration was determined using the Pierce BCA Protein Assay Kit (Thermo) with bovine serum albumin as a standard control. Protein extractions were separated by using SDS-PAGE on 10% polyacrylamide gels, and transferred to polyvinylidene fluoride membranes. After blocking, the membrane was incubated with indicated primary antibodies overnight at 4 °C. After washing for four times, the membranes were incubated in the appropriate HRP-conjugated secondary antibody at 37 °C for 30 min. The protein bands were visualized using chemiluminescent reagents according to the manufacturer’s instructions and quantified using an imageanalyzer Quantity One System.

### Measurement of apoptosis

TdT (terminal deoxynucleotidyl transferase)-mediated dUTP nick-end labeling (TUNEL) assay was performed to detect apoptosis. Following osteogenic differentiation induction treatment, TUNEL assay was performed using *In Situ* Cell Death Detection Kit (Roche), strictly following instructions provided by the manufacturer. Results were expressed as percentage of WT.

### Measurement of protein interaction

Protein interaction of P53 and Bax was evaluated by co-immunoprecipitations (Co-IP) using a Thermo Scientific Pierce Co-Immunoprecipitation Kit. Antibodies for bait proteins (P53) were immobilized covalently using amino-link columns according to manufacturer’s protocol. Lysates were obtained, cleared on agarose resin, and immunoprecipitated according to the protocol. Western blot detection of P53 or Bax was conducted on IP eluates as described above. IP column flow through with no bait antibody was run as an input control.

### Statistical analysis

Statistical analysis was performed by Graph-Pad Prism software and the SPSS software version 15.0. The relationships between variables (BMD, FFA, FBG, lipid peroxidation and mtDNA) were assessed by simple linear regression. Multiple linear regression analyses were also used to determine factors associated with reduced BMD. Animal results were expressed as the means ± SD. Statistical analysis was carried out by one-way analysis of variance (ANOVA) followed by the Newmane Keuls multiple-comparison post hoc test. p value < 0.05 was considered statistically significant.

## Additional Information

**How to cite this article**: Li, J. *et al.* FFA-ROS-P53-mediated mitochondrial apoptosis contributes to reduction of osteoblastogenesis and bone mass in type 2 diabetes mellitus. *Sci. Rep.* **5**, 12724; doi: 10.1038/srep12724 (2015).

## Supplementary Material

Supplementary Information

## Figures and Tables

**Table 1:** Demographic and clinical parameters of type 2 diabetic patients.

**Variables**	**T2D patients**
Age (years)	50.6 ± 12.5
BMI (kg/m^2^)	24.8 ± 2.8
FBG (mmol/L)	10.5 ± 2.2
FFA (mmol/L)	0.69 ± 0.14

All values were shown as means ± SD.

**Table 2:** Multivariate associations of BMD with several factors.

**Variables**	**Coefficient β**	**95% CI**	**Standard error**	***p*****-value**
Femoral neck BMD				
FBG	−0.002	−0.011–0.006	0.004	0.586
FFA	−0.238	−0.316–0.032	0.082	0.006
Lipid peroxidation	−0.415	−0.418–0.002	0.117	0.025
mtDNA	0.421	−0.001–0.432	0.128	0.032
BMI	−0.116	−0.123–0.009	0.033	<0.001
Age	−0.001	−0.016–0.015	0.018	0.813
Lumbar spine BMD				
FBG	0.004	−0.005–0.014	0.005	0.352
FFA	−0.192	−0.232–0.069	0.079	0.009
Lipid peroxidation	−0.284	−0.299–0.014	0.109	0.036
mtDNA	0.317	−0.001–0.332	0.062	0.034
BMI	−0.107	−0.114–0.008	0.026	0.008
Age	0.003	−0.013–0.019	0.023	0.297

**Table 3:** Circulating concentrations of fasting blood glucose, lipid profiles, and bone metabolic markers in db/db mice.

**Variables**	**WT**	**db/db**	**Etomoxir**	**MitoQ**	**PFT-α**
FBG (mmol/L)	4.6 ± 0.8	21.5 ± 3.8*	20.6 ± 4.2	15.3 ± 2.7^#^	22.1 ± 3.9
FFA (mmol/L)	0.57 ± 0.12	1.23 ± 0.15*	1.26 ± 0.11	0.87 ± 0.16#	1.11 ± 0.17
TG (mmol/L)	1.23 ± 0.22	3.16 ± 0.48*	3.37 ± 0.56	2.39 ± 0.26#	3.08 ± 0.39
HDL (mmol/L)	1.83 ± 0.21	1.35 ± 0.17*	1.31 ± 0.16	1.58 ± 0.15#	1.36 ± 0.23
LDL (mmol/L)	0.86 ± 0.15	1.74 ± 0.16*	1.77 ± 0.18	1.12 ± 0.24#	1.69 ± 0.29
ALP (U/L)	132 ± 17	61 ± 18*	95 ± 11#	113 ± 13#	109 ± 14#
Osteocalcin (ng/mL)	93 ± 15	75 ± 8*	86 ± 3#	90 ± 5#	88 ± 6#
TRAP (U/L)	6.1 ± 1.2	12.5 ± 2.3*	8.6 ± 1.3#	7.4 ± 1.8#	7.9 ± 2.4#

All values were shown as means ± SD.

^*^p < 0.05, compared with WT.

^#^p < 0.05, compared with db/db mice.
