# Supplementary material for: FFA-ROS-P53-mediated mitochondrial apoptosis contributes to reduction of osteoblastogenesis and bone mass in type 2 diabetes mellitus
Source: Sci Rep. 2015 Jul 31;5:12724. doi: 10.1038/srep12724 (PMC4521203; doi:10.1038/srep12724)
Supplement: Supplementary Information [file srep12724-s1.doc]

**FFA-ROS-P53-mediated mitochondrial apoptosis contributes to reduction of osteoblastogenesis and bone mass in type 2 diabetes mellitus**

Jun Li^1a^, Wang He^2a^, Bo Liao^3a^, Jingyue Yang^4^*

^1^ Department of Orthopaedic Surgery, Tangdu Hospital, Fourth Military Medical University, Xi’an 710038, China

^2^ Department of Endocrinology, Xi’an NO.1 Hospital, Xi’an 710002, China

^3^ Department of Orthopaedic Surgery, Tangdu Hospital, Fourth Military Medical University, Xi’an 710038, China

^4^ Jingyue Yang, Department of Oncology, Xijing Hospital, Fourth Military Medical University, Xi’an 710032, China

^a^ These three authors contributed equally to this work.

*Corresponding author. Department of Oncology, Xijing Hospital, Fourth Military Medical University, Changle West Road 169, Xi'an 710032, China. E-mail address: jingyue_yang11@163.com (J. Yang)


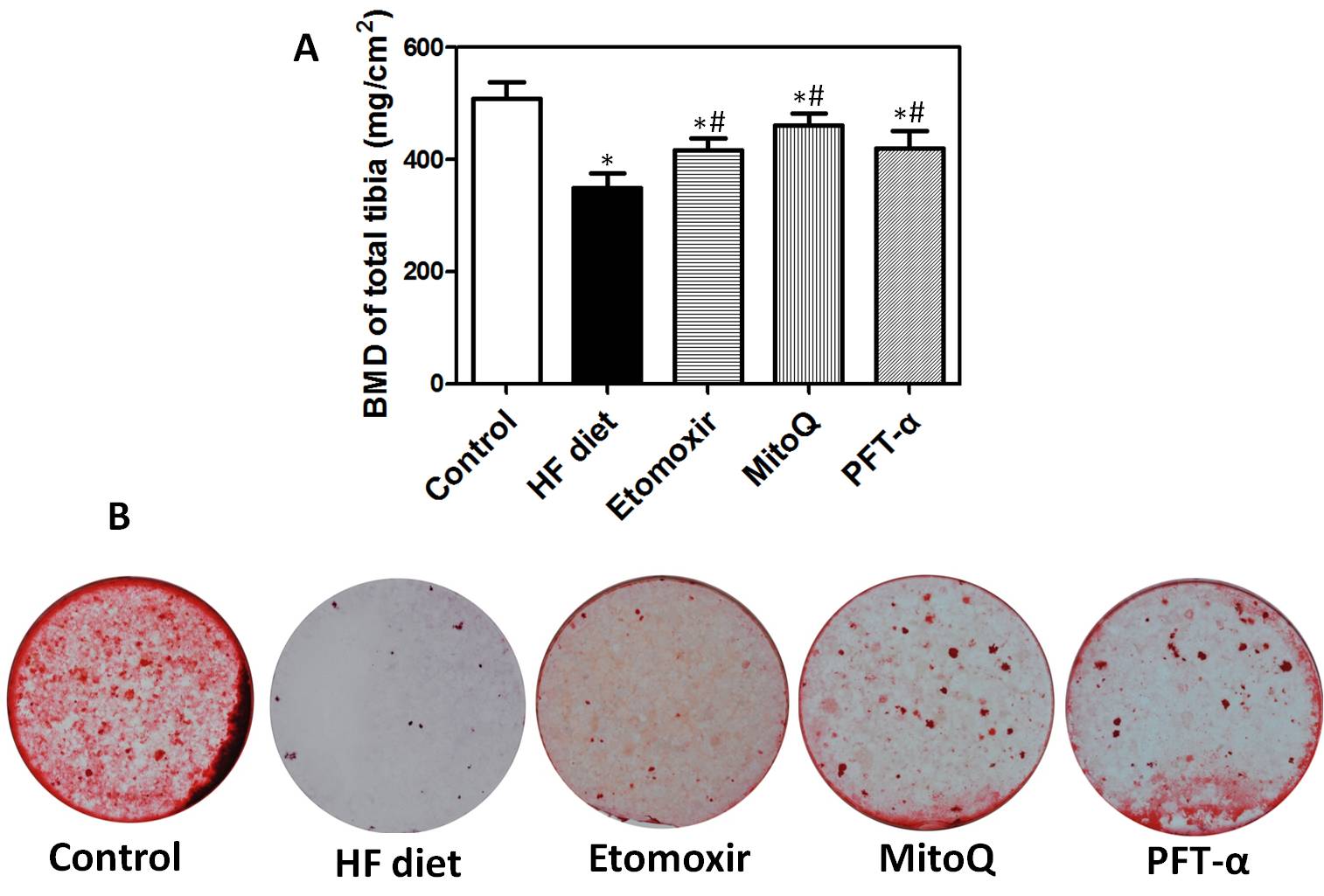


**Supplemental Fig. 1 Alterations of bone mass in mice and osteogenic differentiation of BMSCs in vitro.** HF diet-fed mice were administered with Etomoxir, MitoQ, and PFT-α, for 10 weeks. After that, bone mass density of total tibia were measured (A). BMSCs were isolated and induced to differentiate into osteoblast. Then, osteogenic differentiation was evaluated by alkaline phosphatase staining (B).*p < 0.05, compared with Control. # p < 0.05, compared with HF diet.


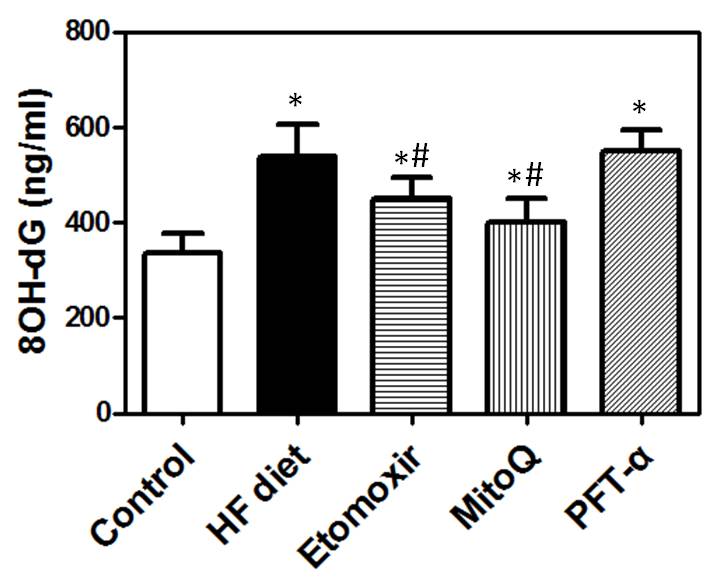


**Supplemental Fig. 2 Alterations of 8OH-dG level in urine in mice fed HF diet.** HF diet-fed mice were administered with Etomoxir, MitoQ, and PFT-α, for 10 weeks. After that, 8OH-dG in urine was determined by commercial kits. *p < 0.05, compared with Control. # p < 0.05, compared with HF diet.


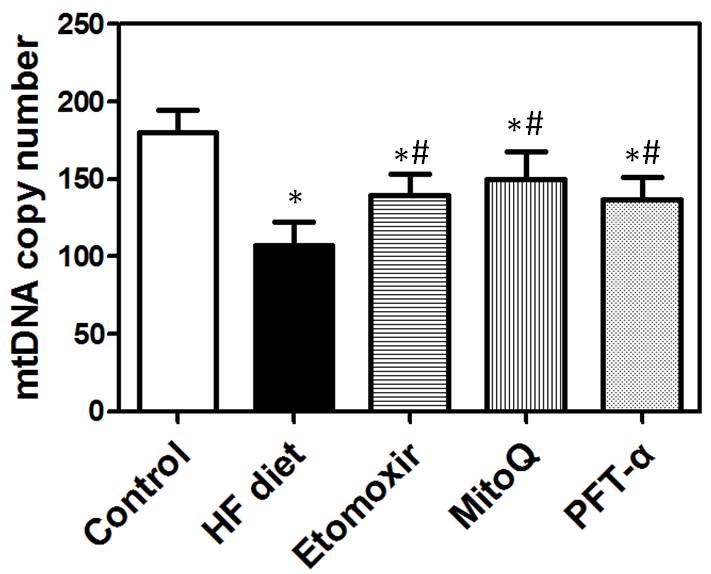


**Supplemental Fig. 3 Alterations of mitochondrial function in differentiated osteoblasts in vitro.** HF diet-fed mice were administered with Etomoxir, MitoQ, and PFT-α, for 10 weeks. After that, osteogenic differentiation of BMSCs was induced in vitro. mtDNA copy number was detected by Real-time PCR. *p < 0.05, compared with Control. # p < 0.05, compared with HF diet.


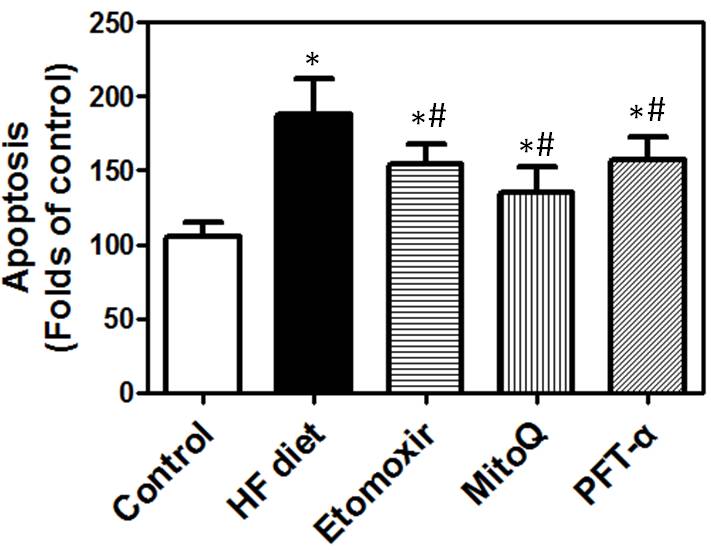


**Supplemental Fig. 4 Alterations of apoptosis in differentiated osteoblasts in vitro.** HF diet-fed mice were administered with Etomoxir, MitoQ, and PFT-α, for 10 weeks. After that, osteogenic differentiation of BMSCs was induced in vitro. TUNEL assay was conducted to evaluate apoptosis. *p < 0.05, compared with Control. # p < 0.05, compared with HF diet.
